# Neonatal abstinence syndrome and infant mortality and morbidity: a population-based study

**DOI:** 10.3389/fped.2024.1394682

**Published:** 2024-07-16

**Authors:** Sarka Lisonkova, Qi Wen, Lindsay L. Richter, Joseph Y. Ting, Janet Lyons, Sheona Mitchell-Foster, Eugenia Oviedo-Joekes, Giulia M. Muraca, Hamideh Bayrampour, Eric Cattoni, Ronald Abrahams

**Affiliations:** ^1^ Department of Obstetrics and Gynaecology, University of British Columbia, Vancouver, BC, Canada; ^2^ School of Population and Public Health, University of British Columbia, Vancouver, BC, Canada; ^3^ Department of Pediatrics, University of Alberta, Edmonton, AB, Canada; ^4^ Northern Medical Program, University of British Columbia, Prince George, BC, Canada; ^5^ Departments of Obstetrics and Gynecology, and Health Research Methods, Evidence, and Impact, McMaster University, Hamilton, ON, Canada; ^6^ Department of Family Practice, Faculty of Medicine, University of British Columbia, Vancouver, BC, Canada

**Keywords:** neonatal abstinence syndrome, infant mortality, follow-up hospitalizations, foster care, maternal characteristics

## Abstract

**Background:**

Infant health among newborns with neonatal abstinence syndrome (NAS) has been understudied. We examined infant mortality and hospitalizations among infants diagnosed with NAS after birth.

**Methods:**

All live births in British Columbia (BC), Canada, for fiscal years from 2004–2005 to 2019–2020, were included (*N* = 696,900). NAS was identified based on International Classification of Diseases, version 10, Canadian modification (ICD-10-CA) codes; the outcomes included infant death and hospitalizations during the first year of life, ascertained from BC linked administrative data. Generalized estimating equation models were used to adjust for maternal factors.

**Results:**

There were 2,439 infants with NAS (3.50 per 1,000 live births). Unadjusted for other factors, infant mortality was 2.5-fold higher in infants with vs. without NAS (7.79 vs. 3.08 per 1,000 live births, respectively) due to increased post-discharge mortality NAS (5.76 vs. 1.34 per 1,000 surviving infants, respectively). These differences diminished after adjustment: adjusted odds ratio (AOR) for infant death was 0.85 [95% confidence interval (CI): 0.52–1.39]; AOR for post-discharge death was 1.75 (95% CI 1.00–3.06). Overall, 22.3% infants with NAS had at least one hospitalization after post-neonatal discharge, this proportion was 10.7% in those without NAS. During the study period, discharge to foster care declined from 49.5% to 20.3% in infants with NAS.

**Conclusion:**

Unadjusted for other factors, infants with NAS had increased post-discharge infant mortality and hospitalizations during the first year of life. This association diminished after adjustment for adverse maternal and socio-medical conditions. Infants with NAS had a disproportionately higher rate of placement in foster care after birth, although this proportion declined dramatically between 2004/2005 and 2019/2020. These results highlight the importance of implementing integrated care services to support infants born with NAS and their mothers during the first year of life and beyond, even though NAS itself is not independently associated with increased infant mortality.

## Introduction

Opioid use has been increasing steadily in North America, and the number of overdose deaths increased by 250% in the last two decades, with a further 30% increase during the COVID-19 pandemic (1–4). British Columbia (BC), Canada, has consistently reported the highest number of overdose deaths in Canada, as the number of deaths due to overdose from illicit drug use exceeded deaths due to COVID-19 in 2020 (5, 6). The incidence of neonatal abstinence syndrome (NAS) also increased in BC from 2.6 per 1,000 live births in 2010–2011 to 4.8 per 1,000 live births in 2019–2020 (7). Simultaneously, there has been a substantial increase in the incidence of NAS reported in the USA, England, Canada, and Western Australia in the past decade (8–10), and some reports suggest a continuation of high rates during the first year of the pandemic (11, 12).

Opioid use in pregnancy can lead to NAS in infants, more recently termed neonatal opioid withdrawal syndrome (NOWS) (13). NAS is a complex disorder primarily affecting the autonomic nervous system and gastrointestinal system of the infant. It is defined by signs and symptoms of withdrawal at 1–3 days after birth (13–15). These clinical manifestations range from tremors and irritability to fever, excessive weight loss, and seizures, requiring prolonged hospital stay (13–15). The long-term effects of opioid use during pregnancy and NAS include an increased risk of infant morbidity, sudden infant death syndrome, psychological abnormalities, and behavioral and mental disorders (15–19), which are influenced by childhood environments with socioeconomic challenges concomitant with substance use, such as family dysfunction, exposure to violent crime, inadequate nutrition, and maternal alcohol or substance use (15).

Despite the continuing increases in NAS, adverse health outcomes during infancy, and in particular in the post-neonatal period, among those diagnosed with NAS have been understudied. In this study, we examined infant mortality and post-discharge infant death and hospitalizations associated with NAS.

## Materials and methods

### Study population and data sources

We carried out a cohort study including all infants born in BC, Canada, between 1 April 2004 and 31 March 2020 (i.e., fiscal years 2004–2005 to 2019–2020), with a follow-up period of 1 year. Information on all live births in BC was obtained from the BC Perinatal Database Registry (BCPDR) (20). The registry captures >99% of all births in the province of BC, with data abstracted from hospital charts after discharge by trained health records staff according to standardized protocols, and validation studies have documented high accuracy of the collected data (21). We excluded infants with a missing mother–infant link, those who were born at <20 and >44 weeks’ gestation, had missing gestational age, and those with birthweight <250 g and >6,500 g or missing birthweight. The follow-up information was obtained through linkage between the BCPDR database (20) and hospitalization files (22), demographic files (23) and Vital Statistics information on deaths (24); all linkages were performed by Population Data BC (20, 22–25) using a unique personal health number assigned to each person under the Canadian universal insurance system.

The BCPDR database provided maternal demographic and pregnancy information, for instance, maternal age; parity; pre-pregnancy morbidity; pre-pregnancy body mass index (BMI); pregnancy complications; mode of delivery; cigarette smoking, alcohol, and drug use during pregnancy; history of prior mental health diagnoses; HIV infection; and infant information, for example, Apgar score at 5 min, infant's sex, and gestational age at birth. “Substance use during pregnancy” indicated a self-reported use of drugs, including heroin/opioids, methadone, stimulants, solvents, or other drugs at any time during pregnancy. “History of prior mental health diagnoses” included any history of mental health illness noted in the medical chart, for instance, depression, anxiety, bipolar disorder, and others (Supplementary Table S1). Gestational age was based on the first trimester ultrasound, when not available, last menstrual period dating was used. BMI (kg/m^2^) was categorized as follows: underweight (<18.5), normal BMI (18.5–24.9), overweight (25.0–29.9), and obesity (≥30.0). BCPDR also includes up to 25 diagnostic [International Classification of Diseases, version 10, Canadian modification (ICD-10-CA) codes] and 20 medical procedure codes (CCI—Canadian Classification of Interventions) related to the delivery and newborn hospitalization, which were used to identify maternal morbidity (e.g., chronic hypertension, chronic diabetes, asthma; Supplementary Table S1), and severe neonatal morbidity. Neonatal morbidity was a composite variable defined as the presence of any of the following: bronchopulmonary dysplasia (BPD), respiratory distress syndrome (RDS), intracranial non-traumatic hemorrhage [e.g., intraventricular hemorrhage (IVH)], periventricular leukomalacia (PVL), retinopathy of prematurity (ROP), neonatal sepsis, convulsions, necrotizing enterocolitis (NEC), and intestinal perforation (Supplementary Table S1). Congenital anomalies were identified by ICD-10-CA codes (all codes starting with “Q”) on infants’ birth hospitalization record.

Linked hospitalization files provided information about post-discharge morbidity and included up to 25 medical diagnostic codes related to each hospitalization. Linked Vital Statistics data included month and year of infant death. Maternal neighborhood at the time of birth was identified by the six-digit maternal postal code, and the demographic files’ (24) neighborhood indices including socioeconomic quintile and rural residential area based on the Statistics Canada Postal Code Conversion files (26). Rural residence was defined as dwellings with <10,000 inhabitants (27), and low and high socioeconomic status (SES) was defined as maternal residences in the lowest and the highest socioeconomic quintile neighborhoods, respectively, in relation to median income in Canada. Average SES (quintile 2–5) was used as a reference category.

### NAS and outcome measures

NAS was identified by the ICD-10-CA code P96.1 for neonatal abstinence syndrome during newborn hospitalization. This is different from the code P96.2, which is used for neonatal withdrawal syndrome due to therapeutic drugs used to treat neonatal conditions.

The primary outcomes were infant death (during the first year after birth) and infant death before and after hospital discharge. Additional outcomes included any post-newborn hospitalization during the first year of life, the total and average number of hospitalizations and median length of stay in the hospital. Furthermore, we examined the frequency of emergency hospitalizations, and the reasons for and conditions diagnosed during hospitalization, for instance, infection and parasitic diseases, malnutrition and nutritional deficiency, any diseases of respiratory system, injuries and poisoning (including maltreatment syndrome), external causes of mortality and morbidity (including motor vehicle accidents and assaults), and injury, burns, and corrosions. These conditions have been found to be associated with NAS in previous studies (18, 19) and were identified by ICD-10-CA codes (Supplementary Table S2) recorded during hospitalizations. We also compared proportions of infants discharged to adoption and foster care services after birth between those with and without NAS among those who survived to hospital discharge, and the changes in discharge destination over the study period.

### Statistical analyses and covariates

Demographic characteristics and risk factors were compared between infants with and without NAS. Infant mortality, as well infant death pre- and post-hospital discharge within the first year of life was compared using rate ratio (RR) and 95% confidence interval (CI). The proportion of infants with at least one hospitalization was described and conditions diagnosed during the hospitalization were compared using RRs and 95% CIs; where low numbers precluded the calculation of RR due to confidentiality reasons, *p*-values were used to assess statistical significance of the differences between infant with vs. without NAS using Fisher’s exact test. Median length of hospital stay was contrasted between both groups using the Mann–Whitney *U*-test.

We used logistic regression with the generalized estimating equations (GEE) method to estimate adjusted odds ratios (AOR) accounting for clustered outcomes in twin infants and other multiples. We used a staged approach of adjusting for distal and proximal covariates; we used first and second stage models for infant death and a third stage-model for post-discharge infant death (Supplementary Table S3). In the first stage, model 1 included pre-pregnancy characteristics: maternal age (15–24 and ≥35vs. 25–34 years), parity (nullipara vs. multipara), pre-pregnancy hypertension, pre-pregnancy diabetes mellitus, asthma, maternal HIV status, prior mental health diagnoses, alcohol use during pregnancy, smoking during pregnancy, *in vitro* fertilization (IVF) conception, pre-pregnancy BMI (underweight, overweight, and obese vs. normal BMI), maternal residence (rural and missing vs. urban area), and SES (low and high SES vs. average SES). We did not adjust for maternal substance use as this is directly linked to the NAS syndrome. In the second stage (model 2), pregnancy characteristics were added as covariates to the models, including plurality (twins/triplets vs. singleton), bleeding during pregnancy at <20 weeks’ gestation, antepartum bleeding/hemorrhage, gestational hypertension, gestational diabetes, placental disorders (also including placenta accrete/increta/percreta), proteinuria during pregnancy, and cesarean delivery. For the GEE regression model of post-discharge infant death (model 3), neonatal characteristics were added in the third stage, including infant's sex, gestational age at birth (20–33 and 34–36 vs. ≥37 weeks), Apgar score at 5 min (<4 vs. 4–10), any congenital anomaly, and severe neonatal morbidity (Supplementary Table S1). Only infants who survived initial birth hospitalization were included in this latter model (i.e., model 3 for post-discharge infant death).

The proportion of missing values exceeded 10% for pre-pregnancy BMI in all women, and for SES and rural/urban indicator in women with infants diagnosed with NAS. These missing values were due to the lack of information on the maternal residence postal code and are likely related to homelessness or transitory maternal residence. Consequently, “missing SES” and “missing rural/urban residence” were used as separate categories in the models. We did not use any multiple imputation strategy to estimate the missing BMI values, as the proportion of missing values exceeded 30% in the NAS group; instead, we included “missing” category for BMI in the model.

All analyses were carried out using SAS 9.4 (SAS Institute Inc., Cary, NC, USA). The study was approved by the University of British Columbia—Children's & Women's Health Centre of BC Research Ethics Board (CW17-0090/H17-00240).

## Results

### Study population

Overall, 697,944 newborns were delivered in BC between 1 April 2004 and 31 March 2020. Records with missing mother–infant link (*n* = 109; 0.02%) were excluded; 707 (0.10%) infants with gestational age <20 and >44 weeks’ or missing information on gestational age were excluded, as well as 228 (0.03%) infants with birthweight <250 or >6,500 g or missing birthweight. The study population included 696,900 live born infants and 685,940 mothers (Table 1). The overall rate of NAS was 3.50 per 1,000 live births.

**Table 1:** Demographic characteristics of mothers of infants with and without NAS: BC, Canada, 2004–2005 to 2019–2020.

Maternal characteristics	With NAS	Without NAS
	*N* = 2,422 (%)	*N* = 683,518 (%)
Maternal age (years)		
≤14	0 (0.0)	52 (0.0)
15–19	50 (2.1)	12,886 (1.9)
20–24	395 (16.3)	72,949 (10.7)
25–29	760 (31.4)	171,451 (25.1)
30–34	712 (29.4)	240,852 (35.2)
35–39	410 (16.9)	148,139 (21.7)
40–60	95 (3.9)	37,188 (5.4)
Pre-pregnancy BMI category		
Underweight	118 (4.9)	29,464 (4.3)
Normal	750 (31.0)	299,410 (43.8)
Overweight	244 (10.1)	105,858 (15.5)
Obese	148 (6.1)	66,748 (9.8)
Missing	1,162 (48.0)	182,038 (26.6)
Parity		
Para 0	845 (34.9)	317,149 (46.4)
Para 1–2	1,077 (44.5)	328,440 (48.1)
Para ≥3	500 (20.6)	37,806 (5.5)
IVF conception	10 (0.4)	15,322 (2.2)
Smoking during pregnancy	1,539 (63.5)	52,263 (7.7)
Alcohol use during pregnancy	219 (9.0)	7,176 (1.1)
Substance use during pregnancy	1,724 (71.2)	20,553 (3.0)
Prior mental health diagnoses	1,064 (43.9)	99,593 (14.6)
Socioeconomic residential quintile		
1 (lowest)	805 (33.2)	137,133 (20.1)
2	456 (18.8)	137,151 (20.1)
3	293 (12.1)	134,821 (19.7)
4	277 (11.4)	135,915 (19.9)
5 (highest)	223 (9.2)	106,879 (15.6)
Missing	368 (15.2)	31,619 (4.6)
Residence		
Urban residence	2,002 (82.7)	618,991 (90.6)
Rural residence	132 (5.5)	40,861 (6.0)
Missing	288 (11.9)	23,666 (3.5)
Chronic hypertension	20 (0.8)	4,773 (0.7)
Chronic diabetes	17 (0.7)	4,142 (0.6)
Chronic hepatic disorders	67 (2.8)	319 (0.1)
Asthma	67 (2.8)	3,896 (0.6)
HIV infection	69 (2.9)	332 (0.1)

See Supplementary Table S1 for more details.

Mothers of infants affected by NAS were more likely to be younger, multiparous with three or more children, 63.5% reported smoking cigarettes during pregnancy, and 71.2% reported substance use (vs. 7.7% and 3.0%, respectively, in women who had infants without NAS). Mothers of infants with NAS had a higher proportion of those who used alcohol during pregnancy, and 43.9% had prior mental health diagnoses. They were less likely to have conceived by *in vitro* fertilization compared with other women (Table 1). Mothers of infants with NAS were also more likely to reside in the lowest SES neighborhoods or have a missing postal code (which can indicate unstable housing or homelessness); they had a higher frequency of chronic hepatic conditions, asthma, and HIV infection (Table 1). With respect to pregnancy complications, they had higher frequency of placental disorders and antepartum hemorrhage and lower rates of gestational diabetes (Table 2).

**Table 2:** Pregnancy and infant characteristics of mothers and infants with and without NAS: BC, Canada, 2004–2005 to 2019–2020.

Pregnancy characteristics	With NAS	Without NAS
	*N* = 2,422 (%)	*N* = 683,518 (%)
Plurality		
Singleton	2,399 (99.1)	672,680 (98.4)
Twins	23 (1.0)	10,691 (1.6)
Triplets	0 (0)	144 (0.0)
Bleeding during pregnancy <20 weeks	37 (1.5)	11,076 (1.6)
Antepartum bleeding/hemorrhage	63 (2.6)	9,421 (1.4)
Gestational hypertension	176 (7.3)	35,984 (5.3)
Gestational diabetes	118 (4.9)	68,622 (10.0)
Placental disorders	30 (1.2)	5,409 (0.8)
Proteinuria	34 (1.4)	8,556 (1.3)
Onset of labor		
Induced	481 (19.9)	149,333 (21.9)
Spontaneous	1,626 (67.1)	431,929 (63.2)
None (cesarean without labor)	315 (13.0)	102,244 (15.0)
Cesarean delivery	800 (33.0)	219,097 (32.1)
Gestational age (weeks)		
20–23	<5	631 (0.1)
24–27	12 (0.5)	1,715 (0.3)
28–33	131 (5.4)	11,044 (1.6)
34–36	574 (23.7)	47,967 (7.0)
37–41	1,681 (69.4)	614,882 (90.0)
42–44	22 (0.9)	7,279 (1.1)
Newborn characteristics	With NAS	Without NAS
	*N* = 2,439 (%)	*N* = 694,461 (%)
Male infant sex	1,217 (49.9)	349,175 (50.3)
Preterm birth <37 weeks	731 (30.0)	68,619 (9.9)
Preterm birth <34 weeks	151 (6.2)	15,643 (2.3)
Birth weight <2,500 g	503 (20.6)	38,834 (5.6)
Apgar score at 5 min <4	18 (0.7)	2,342 (0.3)
Congenital anomaly^a^	286 (11.7)	36,388 (5.2)
Severe neonatal morbidity^b^	168 (6.9)	12,380 (1.8)

^a^ Recognized at birth; all congenital anomalies with ICD-10-CA code “Q”.

^b^ Includes any of the following: BPD, RDS, intracranial non-traumatic hemorrhage (e.g., IVH), PVL, ROP, neonatal sepsis, convulsions, NEC, and intestinal perforation.

See Supplementary Table S1 for more details.

Small cells <5 are not shown due to confidentiality reasons.

Infants with NAS had higher proportion of risk factors including preterm birth (<37 weeks; 20.42% vs. 6.33%), low birth weight (<2.500 g, 20.6% vs. 5.6%), severe neonatal morbidity, and congenital anomalies (Table 2).

### Infant mortality

Infant mortality was 7.79 per 1,000 live births in infants with NAS in contrast to 3.08 per 1,000 live births in infants without NAS (Table 3), with an RR of 2.53 (95% CI: 1.61–3.97). Mortality before discharge was 2.05 vs. 1.75 per 1,000 live births in the NAS group vs. the comparison group (RR = 1.17, 95% CI: 0.49–2.82). While the majority (75.8%) of infants without NAS stayed in the hospital for 1–3 days, majority of infants with NAS stayed for 8 days or more and the median length of stay was significantly different (*p* < 0.001). Almost a third of infants with NAS who survived to discharge were placed in adoptive or foster care (29.4% vs. 0.4% in infants without NAS; Table 3). Infants with NAS were more likely to be transferred to another hospital than infants without NAS. Post-discharge mortality was 5.75 per 1,000 infants who survived to discharge in the NAS group vs. 1.34 per 1,000 infants in the comparison group (without NAS), with an RR of 4.30 (95% CI: 2.54–7.43, Table 3).

**Table 3:** Unadjusted infant mortality and length of stay in the hospital for newborns with and without NAS: BC, Canada, 2004–2005 to 2019–2020.

Infant outcomes	With NAS	Without NAS	Rate ratio (95% CI)
	*N* = 2,439 (%)	*N* = 694,461 (%)	
Death before discharge	5 (0.2)	1,213 (0.2)	1.17 (0.49–2.82)
Infant mortality	19 (0.8)	2,140 (0.3)	2.53 (1.61–3.97)
Length of stay			
1–3 days	314 (12.9)	526,229 (75.8)	0.17 (0.15–0.19)
4–7 days	461 (18.9)	121,347 (17.5)	1.08 (0.99–1.19)
8–30 days	1,074 (44.0)	22,656 (3.3)	13.5 (12.7–14.3)
≥31 days	583 (23.9)	5,148 (0.7)	32.2 (29.6–35.1)
Length of stay (median, Q-range)	16 (6–30)	2 (2–3)	*p*-value <0.001
Infants who survived to discharge	*N* = 2,434 (%)	*N* = 693,248 (%)	
Discharge destination			
Adoption	41 (1.7)	955 (0.1)	12.23 (8.9–16.7)
Foster home	675 (27.7)	2,120 (0.3)	90.7 (83.2–98.9)
Home	1,405 (57.7)	673,934 (97.2)	0.59 (0.56–0.63)
Other hospital	313 (12.9)	16,230 (2.3)	5.49 (4.91–6.14)
Post-discharge death	14 (0.6)	927 (0.1)	4.30 (2.54–7.29)

Proportions may not add up due to missing values; missing values <3% are not presented.

### Post-discharge hospitalizations

The majority of infants did not require hospitalization in the first year of life (after the hospital birth discharge); however, the proportion of infants requiring one or more hospitalization after birth was twofold larger in those with NAS (22.3% vs. 10.7%; Table 4). Among those who were hospitalized, NAS infants had a larger proportion of repeat hospitalizations (i.e., more than one hospitalization) during the first year (6.3% vs. 2.5%) and longer overall hospital stay (median 8 vs. 2 days, *p* < 0.001). Among infants with more than two hospitalizations, the median length of stay was approximately similar in both groups (Table 4). Infants with NAS also had a larger proportion of those who had one or more emergency hospitalization (7.7% vs. 5.1%, Supplementary Table S2).

**Table 4:** Unadjusted post-discharge hospitalizations during the first year after birth for infants with and without NAS: BC, Canada, 2004–2005 to 2019–2020.

Post-discharge hospitalizations during the first year of life	With NAS	Without NAS
	*N* (%)	*N* (%)
All infants	*N* = 2,439	*N* = 694,461
No hospitalization	1,895 (77.7)	620,001 (89.3)
One or more hospitalizations	544 (22.3)	74,460 (10.7)
Infants with post-discharge hospitalization	*N* = 544	*N* = 74,460
Number of hospitalizations		
1	392 (16.1)	57,431 (8.3)
2–3	128 (5.3)	14,444 (2.1)
>3	24 (1.0)	2,585 (0.4)
Age of first admission (months)		
<4	450 (82.7)	57,625 (77.4)
4–12	94 (17.3)	16,835 (22.6)
Total number of hospital days (median, Q-range)	8 (2.5, 23)	2 (1, 6)
<4	172 (31.6)	46,337 (62.2)
4–7	88 (16.2)	12,532 (16.8)
8–14	77 (14.2)	6,115 (8.2)
>14	207 (38.1)	9,476 (12.7)
Total number of hospital days (median, Q-range)	8 (2.5, 23)	2 (1, 6)
Death during hospitalization	<5	253 (0.3)
Infants with more than two hospitalizations	*N* = 47	*N* = 5,802
Median length of stay (for all hospitalizations per infant) (days)		
<4	26 (55.3)	3,372 (58.1)
4 to <7	11 (23.4)	1,181 (20.4)
7 to <14	9 (19.2)	789 (13.6)
≥14	<5	460 (7.9)

Small cells <5 are not shown due to confidentiality reasons.

### Multivariable analyses

In contrast to unadjusted analyses that showed 2.5 times higher odds of infant death [odds ratio (OR) = 2.54, 95% CI: 1.61–4.00; Table 5], adjusted analyses for pre-pregnancy risk factors (model 1) showed no association between NAS and infant death (AOR = 0.85, 95% CI: 0.52–1.39). The absence of any association persisted after additional adjustment for pregnancy complications (model 2, AOR = 0.82, 95% CI: 0.49–1.36) and newborn characteristics (model 3, AOR = 0.70, 95% CI: 0.38–1.29, Table 5).

**Table 5:** Infant mortality before and after discharge from birth hospitalization, unadjusted and adjusted odds ratios, for infants with and without NAS: BC, Canada, 2004–2005 to 2019–2020.

Outcome	OR (95% CI)	AOR1 (95% CI)	AOR2 (95% CI)	AOR3 (95% CI)
Death in the first year	2.54 (1.61–4.00)	0.85 (0.52–1.39)	0.82 (0.49–1.36)	0.70 (0.38–1.29)
Post-discharge death	4.32 (2.55–7.33)	1.75 (1.00–3.06)	1.71 (0.98–3.00)	1.04 (0.56–1.94)

AOR1—model adjusted for pre-pregnancy characteristics of the mother: maternal age, pre-pregnancy BMI, parity, socioeconomic status, rural/urban residence, smoking during pregnancy, prior mental health diagnoses, chronic diabetes, and chronic hypertension. AOR2—additional adjustment for pregnancy complications: bleeding during pregnancy (<20 weeks), antepartum bleeding/hemorrhage, proteinuria, multiple births, and cesarean delivery. AOR3—additional adjustment for newborn characteristics: gestational age at delivery, sex, low Apgar score (<4 at 5 min), congenital anomalies, and severe neonatal morbidity.

The odds of post-discharge infant death (within the first year of life) were fourfold higher in infants with NAS compared with the comparison group (OR = 4.32, 95% CI: 2.55–7.33). However, this association attenuated dramatically and was not statistically significant after adjustment for maternal pre-pregnancy risk factors (model 1, AOR = 1.75, 95% CI: 1.00–3.06), and diminished after additional adjustment for pregnancy complications and newborn risk factors (model 2, AOR = 1.71, 95% CI: 0.98–3.00; and model 3, AOR = 1.04, 95% CI: 0.56–1.94, respectively, Table 5).

### Reasons for hospitalizations

The number of reasons and conditions associated with hospitalizations after newborn hospitalization was larger in infants with NAS (Supplementary Table S3); the largest difference was for the reasons related to “social environment” and/or “problem with primary support” (ICD-10-CA codes Z60 and/or Z63, respectively). Large differences were also observed in “external causes, motor-vehicle accidents” (codes V01-X59, X85-Y39) and “diseases of respiratory system” (ICD codes beginning with “J”). Hospitalizations related to “injuries and poisoning” were also significantly more frequent in infants with NAS.

**Figure 1:**
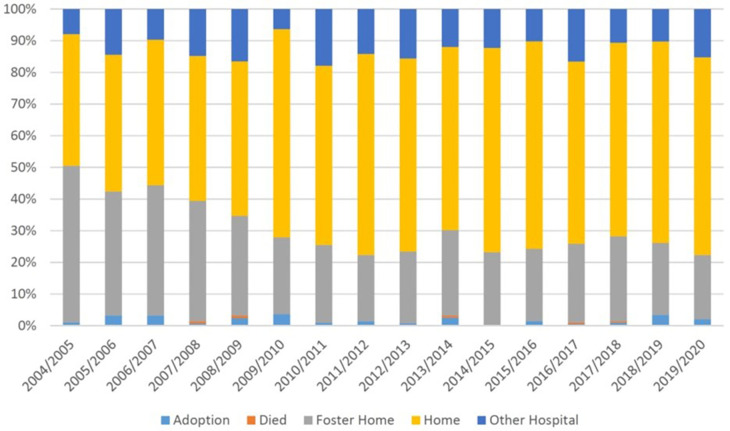
Temporal trends in discharge from neonatal hospitalization among infants with and without NAS: BC, Canada, 2004–2005 to 2019–200.

Mortality prior to discharge remained relatively stable over the study period; however, the proportion of infants with NAS who were placed to foster care declined from 50% in 2004–2005 to 20.3% in 2019–2020, while the proportion of infants discharged home increased (Figure 1, Supplementary Table S4). Similar trends were observed in infants without NAS (Supplementary Table S4).

## Discussion

Our population-based study shows that, unadjusted for other factors, infants with NAS had a 2.5-fold higher infant mortality than infants without NAS and the increase in deaths occurred predominantly after the initial newborn hospitalization with a fourfold elevated risk. This association was no longer present after adjustment for maternal risk factors, including SES and rural/urban/unknown residence, indicating the absence of an independent association between NAS and infant mortality. Rather, the association is due to adverse socioeconomic environment and health conditions of the mother and her infant. Approximately 30% of infants with NAS were discharged to foster care or adoption, which is in sharp contrast with 0.4% of infants without NAS. Unadjusted for other factors, infants with NAS stayed eight times longer in the hospital after delivery, and they were twice as likely to be hospitalized afterward during their first year of life. The largest differences in conditions associated with post-discharge hospitalizations were “problems with social environment and/or primary support”, which were present nearly 35 times more in infants with NAS, “injuries and poisoning” were 3 times more frequent in post-discharge hospitalizations in infants with NAS vs. those without.

Our results are consistent with studies conducted in New South Wales (NSW), Australia, which examined the mortality among children with NAS over a 13-year follow-up period (18, 19), showing a (unadjusted) higher rate of overall mortality (1.4%) in children with NAS. Most deaths in NSW occurred in the post-neonatal period (between 28 days and 1 year of life) with a rate 2.3 times higher in children with vs. without NAS. As in our study, the association diminished after controlling for maternal characteristics (e.g., age, smoking, antenatal care), SES, and rural residence in children born preterm (23–36 weeks gestation); however, it persisted in those born at term. Increased post-neonatal mortality was observed also in studies from the US (28). In contrast to post-neonatal mortality, our study shows increased survival of infants with NAS in the neonatal period (or to hospital discharge), which has been consistently described in the literature (9, 17, 18, 27) as an unexpected phenomenon yet awaiting adequate explanation (29, 30). One possible explanation includes “immortal time bias.” NAS is typically diagnosed 1–2 days after birth; however, most neonatal deaths occur in the first day after birth; thus, in some infants with NAS, diagnosis is never made in those who died very soon after birth. This bias is not present in comparisons of infants who survived to hospital discharge, showing higher unadjusted mortality among infants with NAS.

In addition to substance use, mothers of infants with NAS had a high proportion of those who smoked and used alcohol during pregnancy. Furthermore, 44% indicated prior mental health diagnoses, alongside low SES and an unknown residence. Prior studies show that these mothers have also higher rates of severe maternal morbidity and mortality (10, 31), all of which indicates a complex adverse social environment and medical conditions requiring multidisciplinary support for an extended period after birth.

In our study, we observed that one in four infants with NAS were placed in adoption or foster care after birth, suggesting that the circumstances of the mother and/or her family were not conducive to healthy infant development. However, the time of childbirth often presents an opportune moment to intervene in these mothers’ lives, as the new baby heightens their motivation and desire for improvement in health and social conditions (32–34). Mother–infant rooming-in and bonding are recognized as the most successful post-delivery strategies yielding positive outcomes (13, 33). Therefore, these practices should be extensively utilized during delivery hospitalization, and the transition from the hospital stay should prioritize the utilization of longer-term supporting programs for these vulnerable mothers and their infants. It is encouraging that the proportion of infants with NAS who were placed in adoption/foster care declined in our study from 50% in 2004–2005 to approximately 22% in 2019–2020. This observation is in contrast with the US data showing that the use of child welfare services has increased for infants whose parents used opioids in the US, mirroring the rise of the opioid epidemic over the past decade (35).

Large study population and consistently collected detailed demographic and clinical data are some of the strengths of our study. The data collection is subjected to regular accuracy checks (20, 21). We had information on nearly all live births in BC, including infants born out-of-hospital who may have been subsequently hospitalized because of NAS or other conditions requiring hospitalization, minimizing selection bias. The ICD-10 code for NAS has shown a high accuracy in a large validation study in the United States using hospital administrative data with a positive predictive value of 98.2% (36).

Our study has also several limitations. First, clinical signs of NAS may be subjective, and thus the diagnosis may be missed in infants with milder symptoms. Fetal exposure to opioids depends on the amount and purity of the drugs taken by the mother and the individual kinetics of metabolism and placental drug transfer; thus, NAS occurs in 60%–80% of *in utero* opioid-exposed neonates (37, 38). *In utero* antidepressant and alcohol use can also result in withdrawal symptoms similar to NAS and may have been diagnosed as such (39). However, prior studies have shown that the proportion of NAS resulting from these exposures is relatively small (40). In BC, infants are diagnosed and cared for according to practice guidelines developed by Perinatal Services BC (41, 42). Newborn drug testing is not routine; however, it may be indicated based on maternal history and/or clinical signs. Second, coding errors and omissions could occur in the hospitalization database and in Vital Statistics data, which may have led to an underestimation of infant mortality. We expect the degree of such underestimation to be similar (i.e., non-differential) between infants with and without NAS, which can lead to bias toward null. Third, we did not have a link between subsequent births to the same mother, and therefore could not account for the correlation in outcomes among siblings. Fourth, behavioral factors, including cigarette smoking, alcohol, and substance use, and prior mental health diagnoses, were self-reported to a healthcare provider, and thus subject to underreporting. Fifth, we lacked information on clinical details about infants diagnosed with NAS, including treatment modalities. In addition, specific reasons for hospital transfers were not available for this analysis; however, transfers typically occurred to a higher-level hospital. Finally, we did not have important information on factors that can affect post-discharge survival rates, including, for instance, marital status, family support, access to healthcare, and race/ethnicity (e.g., indigenous people status). We assumed a prognostic perspective on infant mortality without specifically examining causal association and causal pathways between NAS and infant death. We did not have reliable information on the causes of infant death; however, the conditions associated with hospitalizations indicated that the frequency of problems with social environment and primary support was about 35-fold higher in children with NAS.

## Conclusion

In conclusion, our study shows that infants with NAS have elevated unadjusted mortality and post-discharge hospitalization rates after birth. The association between NAS and post-discharge mortality was confounded by extraneous factors, including maternal residence, low SES, and mental health conditions, indicating the absence of an independent association between NAS and infant mortality. Nevertheless, these infants and their mothers need support after birth and discharge from the hospital. Pregnancy and childbirth present a unique opportunity for compassionate multidisciplinary care to improve health and wellbeing of vulnerable mothers and infants. Post-discharge support is crucial to help vulnerable families and babies, including those with NAS, to ensure the best possible health and wellbeing trajectory for all at-risk infants.

## Data Availability

The data analyzed in this study is subject to the following licenses/restrictions: Access to data provided by the Data Stewards is subject to approval but can be requested for research projects through the Data Stewards or their designated service providers. The following data sets were used in this study: BC Perinatal Database, Hospital Separations, BC Consolidation file, and BC Vital Statistics. Population Data BC. Data Extract. MOH; 2017. You can find further information regarding these data sets by visiting the PopData project webpage at: https://my.popdata.bc.ca. Requests to access these datasets should be directed to https://www.popdata.bc.ca/. All inferences, opinions, and conclusions drawn in this publication are those of the authors, and do not reflect the opinions or policies of the Data Stewards.
