# Supplementary material for: Neonatal abstinence syndrome and infant mortality and morbidity: a population-based study
Source: Front Pediatr. 2024 Jul 16;12:1394682. doi: 10.3389/fped.2024.1394682 (PMC11286564; doi:10.3389/fped.2024.1394682)
Supplement: Supplementary file 1 [file Datasheet1.pdf]

Supplementary Tables:

Table S1: Details for maternal and infant risk factors and clinical characteristics. Maternal risk factors as defined by BC Perinatal Database Registry*, Canada.

Risk Factor	Definition
Smoking during pregnancy	Care provider documented in the medical chart that mother reports smoking cigarettes at any time during pregnancy. Includes women who stopped or reduced smoking during pregnancy.
Alcohol use during pregnancy	Care provider documented in the medical chart alcohol as a risk in pregnancy. Alcohol use prior to the woman knowing she was pregnant is not included.
Substance use during pregnancy	A self-reported use of use of drugs, including heroin/opioids, methadone, stimulants, solvents or other drugs at any time during pregnancy.
Prior mental health diagnoses	Any history of mental health illness noted in the medical chart, for instance, depression, anxiety, bipolar disorder, and other.

*BC Database Registry Report

Maternal and neonatal morbidity and ICD-10-codes**

Diagnosis	ICD-10-CA code
**Maternal morbidity**	
Chronic hypertension	O10, O11, I11-I15
Preeclampsia	O14
Eclampsia	O15
Chronic Diabetes	O24.5-O24.7, E10, E11
Chronic Hepatic Disorders	B18, K70, K71, K72.1, K73-K77
Asthma	J45, J46
HIV infection	B20-B24, O98.7, Z21
Placental disorders	O43-O45
Placenta previa	O44
Placental abruption	O45
Adherent placenta (accreta/increate/percreta)	O43.2
**Neonatal morbidity**	
Intracranial hemorrhage (non-traumatic) of newborn	P52
Periventricular leukomalacia	P91.2
Convulsions of newborn	P90
Retinopathy of prematurity	H35.1
Respiratory distress syndrome	P22.0
Bronchopulmonary dysplasia	P27.1

Pneumothorax	P25.1
Necrotizing enterocolitis	P77
Perinatal intestinal perforation	P78.0
Sepsis	P36

** These codes were abstracted from maternal and newborn medical records into the BC Perinatal Database Registry. These codes were used to identify clinical conditions presented in the manuscript.

Table S2: Unadjusted post-discharge emergency hospitalizations during the first year after birth, infants with and without NAS; British Columbia, Canada, 2011/12-2019/20.

Post-discharge emergency hospitalizations during the first year of life	With NAS N (%)	Without NAS N (%)
**All Infants**	**N=2439**	**N=694461**
No emergency hospitalization	2250 (92.3)	658726 (94.9)
One or more	189 (7.7)	35735 (5.1)
**Infants with one or more emergency hospitalizations**	**N=189**	**N=35735**
Frequency		
1	163 (86.2)	30764 (86.1)
2-3	22 (11.6)	4595 (12.9)
>3	<5	376 (1.0)
Age at the first emergency hospitalization		
<4 months	108 (57.1)	23207 (64.9)
4-12 months	81 (42.9)	12528 (35.1)
Death during the emergency hospitalization	0 (0.0)	51 (0.1)

Small cells <5 are not shown due to confidentiality reasons.

Table S3: Conditions associated with post-discharge hospitalizations in infants with and without NAS at birth; British Columbia, Canada, 2004/05-2019/20.

**Diagnoses associated with post-discharge hospitalizations and ED visits during the first year of life**	**With NAS**		**Without NAS**		**Relative risk (95% CI)**	**p-value**
	N=2434	Rate per 1000	N= 693248	Rate per 1000		
**Hospitalizations**						
Infections (A00-B99)	23	9.45	2824	0.41	2.32 (1.54-3.5)	<.0001
Malnutrition (E40-64)	1-4	0.41-2.15	176	0.03	-	0.129
Diseases of Respiratory System (J)	54	22.19	5180	0.75	2.97 (2.27-3.88)	<.0001
Injuries, poisoning (S, T0-T7, T9)	7	2.88	646	0.09	3.09 (1.47-6.50)	0.002
Maltreatment syndrome (T74)	1-4	0.41-2.15	37	0.01	-	0.125
External causes, motor-vehicle accidents (V01-X59, X85-Y39)	18	7.40	2135	0.31	2.40 (1.51-3.82)	<.001
Adverse effects of medications , sequelae (Y4 -Y8)	7	2.88	2296	0.33	0.87 (0.41-1.82)	0.709
Social environment (Z60) or Problem with primary support (Z63)	13	5.34	107	0.02	34.6 (19.5-61.5)	<.0001
Congenital anomalies (Q)	62	25.47	9497	1.37	1.86 (1.45-2.39)	<.0001
Conditions originating in prenatal period (P)	308	126.54	26229	3.78	3.34 (2.99-3.74)	<.0001
**Emergency hospitalizations***	N=2434		N= 693248			
Infections (A00-B99)	33	13.56	7373	1.06	1.27 (0.91-1.79)	0.161
Malnutrition (E40-64)	0	0.00	82	0.01	-	0.999
Diseases of Respiratory System (J)	96	39.44	14855	2.14	1.84 (1.51-2.25)	<.0001
Injuries, poisoning (S, T0-T7, T9)	11	4.52	1773	0.26	1.77 (0.98-3.20)	0.056
Maltreatment syndrome (T74)	1-4	0.41-2.15	105	0.02	-	0.310
External causes, motor-vehicle accidents (V01-X59, X85-Y39)	15	6.16	1979	0.29	2.16 (1.30-3.59)	0.002
Adverse effects of medications , sequelae (Y4 -Y8)	1-4	0.41-2.15	1094	0.16	-	0.601
Social environment (Z60) or Problem with primary support (Z63)	1-4	0.41-2.15	63	0.01	-	0.201
Congenital anomalies (Q)	12	4.93	2786	0.4	1.23 (0.70-2.16)	0.478
Conditions originating in prenatal period (P)	19	7.81	7152	1.03	0.76 (0.48-1.19)	0.221

P-values based on chi-square or Fishers exact test

NOTE: infants could have more than one conditions diagnosed during hospitalization

* unplanned hospitalizations

Small cells <5 are not shown due to confidentiality reasons, presented as a count 1-4.

Table S4: Temporal trends in discharge from neonatal hospitalization among infants with and without NAS; British Columbia, Canada, 2004/05-2019/20.

Fiscal Year	Infants with NAS %					Infants without NAS %					Rate Ratio (NAS vs no NAS)				
	Adoption	Died	Foster Home	Home	Other Hospital	Adoption	Died	Foster Home	Home	Other Hospital	Adoption	Died	Foster Home	Home	Other Hospital
2004/2005	0.99	0.00	49.50	41.58	7.92	0.21	0.17	0.35	97.04	2.24	4.7	0.0	141.4	0.4	3.5
2005/2006	3.20	0.00	39.20	43.20	14.40	0.18	0.20	0.43	96.86	2.33	17.8	0.0	91.2	0.4	6.2
2006/2007	3.23	0.00	41.13	45.97	9.68	0.19	0.18	0.48	96.86	2.28	17.0	0.0	85.7	0.5	4.2
2007/2008	0.70	0.70	38.03	45.77	14.79	0.17	0.20	0.43	96.77	2.44	4.1	3.5	88.4	0.5	6.1
2008/2009	2.36	0.79	31.50	48.82	16.54	0.19	0.19	0.38	96.88	2.36	12.4	4.2	82.9	0.5	7.0
2009/2010	3.60	0.00	24.32	65.77	6.31	0.16	0.15	0.28	97.12	2.30	22.5	0.0	86.9	0.7	2.7
2010/2011	0.94	0.00	24.53	56.60	17.92	0.14	0.16	0.28	97.14	2.28	6.7	0.0	87.6	0.6	7.9
2011/2012	1.35	0.00	20.95	63.51	14.19	0.13	0.20	0.31	96.96	2.39	10.4	0.0	67.6	0.7	5.9
2012/2013	0.71	0.00	22.70	60.99	15.60	0.11	0.19	0.30	97.11	2.30	6.5	0.0	75.7	0.6	6.8
2013/2014	2.52	0.63	27.04	57.86	11.95	0.11	0.19	0.29	97.13	2.28	22.9	3.3	93.2	0.6	5.2
2014/2015	0.00	0.00	23.23	64.52	12.26	0.12	0.17	0.30	96.86	2.55	0.0	0.0	77.4	0.7	4.8
2015/2016	1.46	0.00	22.82	65.53	10.19	0.07	0.13	0.26	97.08	2.46	20.9	0.0	87.8	0.7	4.1
2016/2017	0.49	0.49	24.88	57.56	16.59	0.08	0.15	0.27	97.13	2.37	6.1	3.3	92.1	0.6	7.0
2017/2018	0.93	0.46	26.85	61.11	10.65	0.10	0.17	0.22	97.35	2.16	9.3	2.7	122.0	0.6	4.9
2018/2019	3.41	0.00	22.73	63.64	10.23	0.12	0.17	0.23	97.13	2.36	28.4	0.0	98.8	0.7	4.3
2019/2020	2.03	0.00	20.30	62.44	15.23	0.14	0.18	0.10	97.28	2.29	14.5	0.0	203.0	0.6	6.7

Note: Proportions of live born infants with NAS and without NAS.
